# An Unusual Case of Fusarium Endophthalmitis: The Importance of Early Diagnosis and Treatment

**DOI:** 10.7759/cureus.85642

**Published:** 2025-06-09

**Authors:** Jeevakanthi Rajendran, Jia Wei Chai, Pey Yih Ng, Chee Yik Chang

**Affiliations:** 1 Medicine, Hospital Sultanah Nora Ismail, Batu Pahat, MYS; 2 Ophthalmology, Hospital Sultanah Nora Ismail, Batu Pahat, MYS; 3 Infectious Diseases, Hospital Sultanah Aminah, Johor Bahru, MYS

**Keywords:** fungal endophthalmitis, fusarium species, intravitreal injections, pars plana vitrectomy, voriconazole therapy

## Abstract

Fusarium endophthalmitis is a rare but devastating ocular infection, often associated with trauma or systemic immunosuppression. It is caused by Fusarium species, a group of filamentous fungi that are widely found in soil and plant material. The infection is characterized by rapid progression and poor visual prognosis if not promptly treated. We present an interesting case of Fusarium endophthalmitis in an immunocompetent elderly male with no prior ocular illness. Early diagnosis and prompt administration of antifungal therapy, including intravitreal amphotericin B and systemic voriconazole, along with surgical intervention, contributed to a favorable clinical outcome.

## Introduction

Fusarium species are ubiquitous filamentous fungi widely distributed in soil, plant debris, and water sources. They are recognized primarily as plant pathogens but have emerged as opportunistic human pathogens capable of causing a spectrum of clinical infections [1,2].

Invasive fusariosis predominantly affects immunocompromised individuals, particularly those with hematological malignancies, neutropenia, or those receiving immunosuppressive therapy. The disease may present as a disseminated infection affecting the skin, lungs, sinuses, and occasionally the eyes [2]. Ocular involvement most commonly arises via direct inoculation following trauma or surgery, leading to fungal keratitis and, in some cases, exogenous endophthalmitis. In contrast, endogenous Fusarium endophthalmitis, resulting from hematogenous spread, is exceedingly rare and typically occurs in the setting of systemic infection in severely immunocompromised patients [3]. Here, we report a rare case of Fusarium endophthalmitis in an elderly, immunocompetent patient with no recent history of trauma or ocular surgery.

## Case presentation

A 73-year-old male farmer with no known medical or ocular illness presented to the ophthalmology clinic with a three-week history of progressive right eye pain and visual decline. He denied any history of ocular trauma, uveitis, recent intraocular surgery, or systemic illness. Notably, his daily activities involved extensive exposure to soil due to farming. He also reported previous ownership of a cat and occasional contact with cat faeces from a neighbour’s pet.

**Figure 1:**
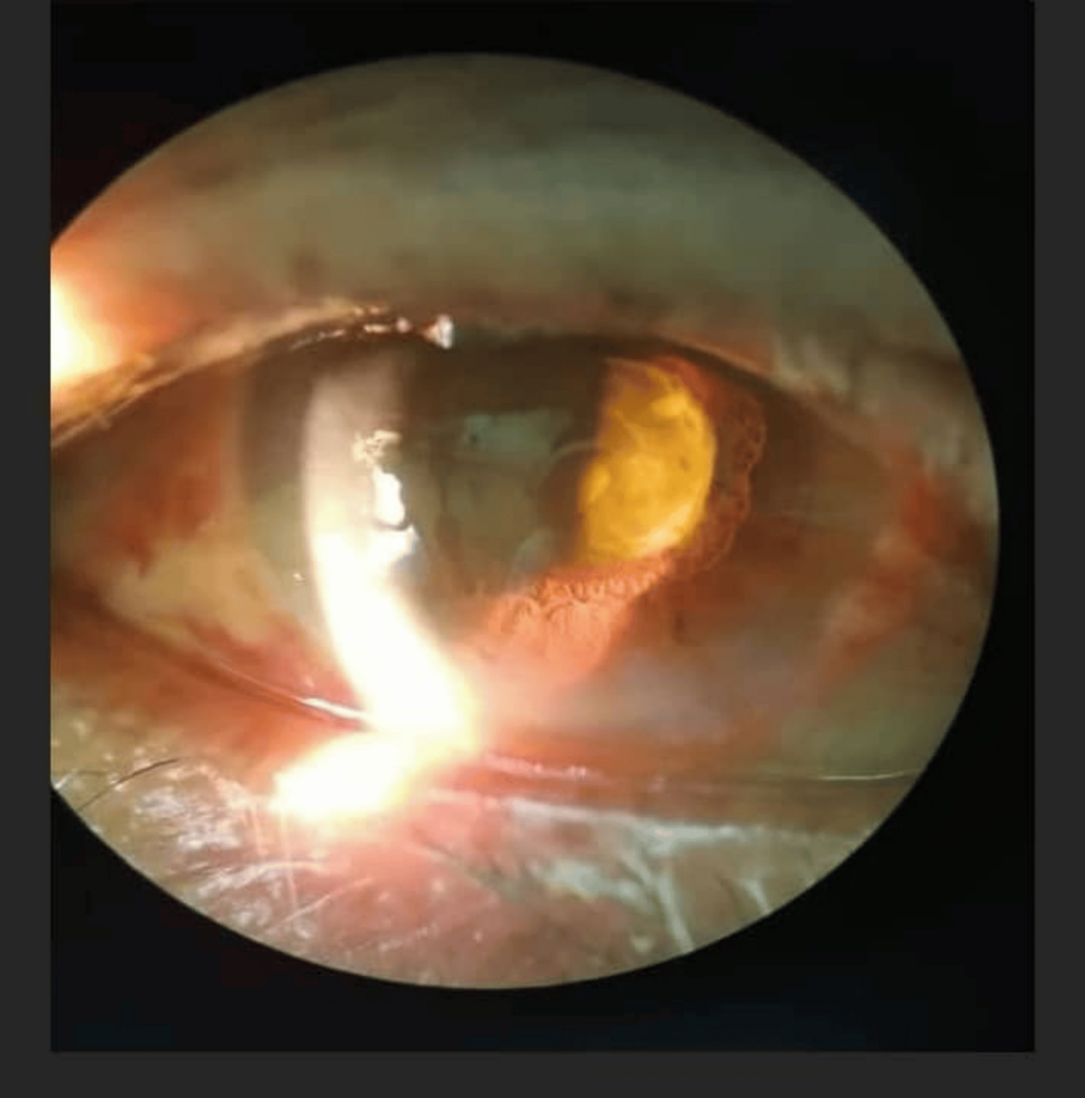
Slit lamp examination of the right eye showing mild conjunctival hyperemia with clear cornea, as well as pronounced vitritis visible through the dilated pupil.

**Figure 2:**
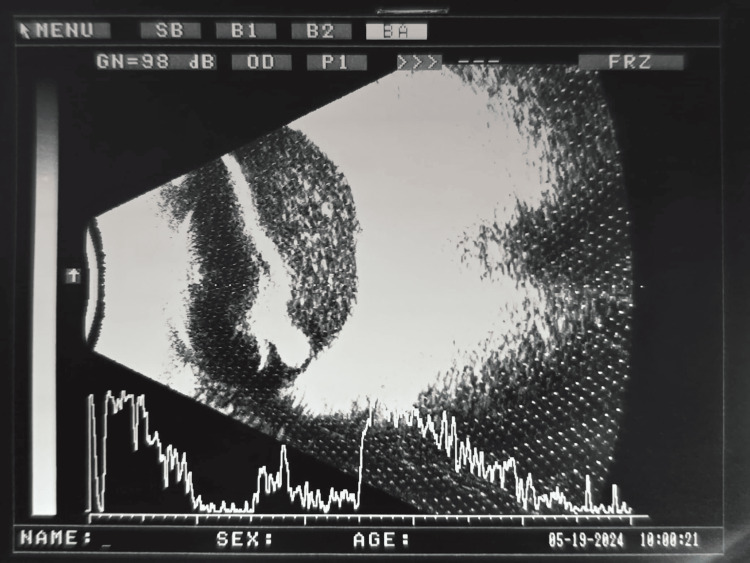
B-scan of the right eye showing dense vitritis with suspicious retinal thickening.

Detailed ophthalmic examination of the right eye revealed dense vitritis and an associated chorioretinal lesion consistent with chorioretinitis (Figures 1, 2). Otherwise, the anterior segment examination of right eye was unremarkable except for the presence of anterior cells 2+ with nucleus sclerosis 1+. The left eye examination was unremarkable. Blood investigations were within normal limits. Serological testing demonstrated reactive *Toxoplasma gondii* IgG but negative IgM. However, he exhibited no systemic features suggestive of active toxoplasmosis. Empiric treatment was initiated with oral co-trimoxazole and prednisolone for presumed ocular toxoplasmosis.

Despite therapy, the patient’s symptoms worsened, prompting hospital admission for evaluation of suspected endogenous endophthalmitis. He underwent multiple intravitreal taps and was treated empirically with intravitreal antibiotics. Vitreous cultures later yielded the growth of Fusarium species. A systemic workup, including HIV, urine and blood cultures, chest radiography, and abdominal imaging, revealed no evidence of disseminated infection or hematological malignancy. His fasting blood glucose levels were within normal range, ruling out occult diabetes mellitus.

On further history taking, the patient recalled a remote episode of minor right eye trauma sustained while farming approximately two years prior. However, there were no clinical signs suggestive of prior penetrating injury during the current examination. Based on the microbiological findings, antifungal therapy with oral voriconazole was initiated alongside intravitreal amphotericin B injections. He subsequently underwent pars plana vitrectomy and intravitreal amphotericin B injection, during which retinal abscesses and a macular scar were noted. Postoperatively, the patient showed clinical improvement, and follow-up vitreous cultures were negative.

He was discharged in stable condition with a planned three-month course of oral voriconazole. During the admission, *Bartonella henselae* serology was sent, and the results are as follows: *B. henselae* IgM: positive and IgG: positive (titre >1:512). The results indicated the possibility of concurrent neuroretinitis due to *B. henselae*; therefore, a six-week course of oral doxycycline was prescribed. The right eye condition remained stable during the follow-up visit at the ophthalmology clinic.

## Discussion

Fusarium species are filamentous fungi that are ubiquitous in the environment, particularly in soil, organic debris, and water sources. They are increasingly recognized as opportunistic pathogens in humans [1,2]. While superficial infections such as keratitis and onychomycosis are relatively common, invasive fusariosis, including endogenous fungal endophthalmitis, is rare and typically occurs in immunocompromised individuals, such as those with hematological malignancies, neutropenia, or recipients of organ or stem cell transplants [2].

Endogenous Fusarium endophthalmitis in immunocompetent individuals is exceedingly rare, with only a limited number of cases reported in the literature. In most cases, ocular involvement arises from direct inoculation following trauma or surgery [4,5]. However, in our patient, there was no recent history of ocular trauma, surgery, or systemic illness, making this presentation highly unusual. The only remote risk factor identified was a minor eye injury that occurred two years earlier while farming, highlighting how Fusarium may persist in a latent form and later reactivate under favorable conditions.

Fusariosis carries a significant mortality risk. The overall mortality rate is estimated at 37%, but this increases to as high as 83% in disseminated cases [6]. Ocular involvement, particularly endophthalmitis, poses a therapeutic challenge due to the aggressive nature of the infection, diagnostic delay, and intrinsic resistance of Fusarium to many antifungal agents.

Previous studies have demonstrated successful treatment of Fusarium endophthalmitis using systemic voriconazole, either alone or in combination with amphotericin B, along with intravitreal antifungal therapy [7-9]. Voriconazole is the preferred agent due to its good intraocular penetration and activity against Fusarium spp. Intravitreal injections of amphotericin B or voriconazole are commonly used to achieve high local drug concentrations [10]. 

A retrospective study conducted at a single centre found that 10 out of 159 patients with Fusarium keratitis progressed to endophthalmitis. All patients received a combination of topical and systemic antifungal therapy. Despite this, clinical outcomes were poor - four required penetrating keratoplasty, two underwent enucleation, two needed both penetrating keratoplasty and pars plana vitrectomy, and one patient progressed to phthisis bulbi [11]. 

The present case further highlights the diagnostic complexity of fungal endophthalmitis. Early ophthalmologic assessment, prompt identification of fungal pathogens, and timely initiation of appropriate systemic and intravitreal antifungal therapy are essential for effective management.

## Conclusions

Fusarium endophthalmitis is an exceptionally rare and vision-threatening condition, particularly in immunocompetent individuals. This case illustrates that even in the absence of risk factors, Fusarium infection should be considered in patients presenting with progressive intraocular inflammation unresponsive to conventional therapy.
